# Long-lived polarization memory in the electronic states of lead-halide perovskites from local structural dynamics

**DOI:** 10.1038/s41467-018-06009-3

**Published:** 2018-08-30

**Authors:** Jasmine P. H. Rivett, Liang Z. Tan, Michael B. Price, Sean A. Bourelle, Nathaniel J. L. K. Davis, James Xiao, Yatao Zou, Rox Middleton, Baoquan Sun, Andrew M. Rappe, Dan Credgington, Felix Deschler

**Affiliations:** 1 Cavendish Laboratory, University of Cambridge, JJ Thomson Avenue, CB3 0HE Cambridge, UK; 2 Department of Chemistry, University of Pennsylvania, Philadelphia, PA 19104 USA; 3 School of Chemical and Physical Sciences, Victoria University of Wellington, Wellington, 6140 New Zealand; 4 Institute of Functional Nano & Soft Materials (FUNSOM), Soochow University, 199 Ren’ai Road, 215123 Suzhou, People’s Republic of China; 5 Department of Chemistry, University of Cambridge, Lensfield Road, CB2 1EW Cambridge, UK

## Abstract

Anharmonic crystal lattice dynamics have been observed in lead halide perovskites on picosecond timescales. Here, we report that the soft nature of the perovskite crystal lattice gives rise to dynamic fluctuations in the electronic properties of excited states. We use linear polarization selective transient absorption spectroscopy to study the charge carrier relaxation dynamics in lead-halide perovskite films and nanocrystals. We find that photo-excited charge carriers maintain an initial polarization anisotropy for several picoseconds, independent of crystallite size and composition, and well beyond the reported timescales of carrier scattering. First-principles calculations find intrinsic anisotropies in the transition dipole moment, which depend on the orientation of light polarization and the polar distortion of the local crystal lattice. Lattice dynamics are imprinted in the optical transitions and anisotropies arise on the time-scales of structural motion. The strong coupling between electronic states and structural dynamics requires a unique interpretation of recombination and transport mechanisms.

## Abstract (web summary)

Anharmonic ultrafast structural dynamics are expected in lead halide perovskites due to their soft nature. Here Rivett et al. show that these dynamics lead to picosecond-long polarization anisotropy of photo-carriers in several halide perovskites, orders of magnitude slower than in conventional semiconductors.

## Introduction

Metal-halide perovskites of composition ABX_3_ (where A = CH_3_NH_3_ or Cs, B = Pb, and X = I, Br, or Cl) are a recent class of solution-processable crystalline semiconductors. Perovskite solar cell power conversion efficiencies now exceed 20%, and efficient light emitting devices with high color purity have been reported^1^. Their excellent photovoltaic performance derives from the perovskites’ band-like semiconducting behavior, with high absorption coefficients^2^ in the range of 10^5^ cm^−1^, combined with long carrier lifetimes^3,4^. These materials lie between the extremes of highly-ordered, crystalline semiconductors, which can exhibit ballistic charge transport, and disordered, molecular semiconductors, where strong electron–phonon coupling leads to highly localized excited states. Lattice distortions, such as lattice phonons, and structural dynamics are key to understand the physics of these materials^5,6^. Recent reports have studied lead halide perovskite lattice dynamics on ultrafast timescales using diffraction^7,8^, Raman^9^, and transient optical Kerr-effect^10,11^ experiments. Further, the combination of strong spin-orbit coupling and local electric fields generated in a non-centrosymmetric crystal lattice^12–14^ can give rise to Rashba-type symmetry breaking in carrier momentum space^15^. Signatures of the Rashba effect have been detected in lead halide perovskites at low temperatures, however the range of reported splitting energies is large (4–240 meV)^16,17^.

Here, we use linear polarization selective transient absorption spectroscopy (LP-TA) to investigate how the reported crystal dynamics affect the electronic states occupied by photoexcited carriers in lead halide perovskite thin films with organic and inorganic A-site cations (CH_3_NH_3_PbX_3_, X = I, Br, CsPbBr_3_) and nanocrystals (CsPbI_3_) at room temperature. This method is sensitive to the coupling between the optical polarization vector of the absorbed light and the transition dipole matrix (TDM) element of the electronic states, which allows us to probe optical anisotropies in the excited state population. Optical alignment upon linearly-polarized excitation occurs in a range of semiconductors, with a variety of underlying causes. In GaAs, the dependence of the optical TDM on the angular momentum of the electronic wave functions imprints a short-lived anisotropic carrier momentum distribution on the excited state population^18–21^. This is lost through femtosecond carrier–carrier scattering. By contrast, optical alignment in molecular materials, with more localized excitonic states, may arise from an alignment of the TDM with physical structure^22–25^. Loss of polarization memory in this case arises from physical reorientation of the photoexcited molecule or diffusion of the excited state to regions with different dipole matrix orientation. Here, we show that lead halide perovskites lie between these two extremes. Their soft structural nature allows dynamic symmetry breaking of the delocalized electronic states, preserving optical alignment far beyond the timescale of momentum-scattering events. Optical alignment is lost on the timescales of local structural reorientation rather than diffusion.

## Results

### Linear polarization selective transient absorption

**Fig. 1:**
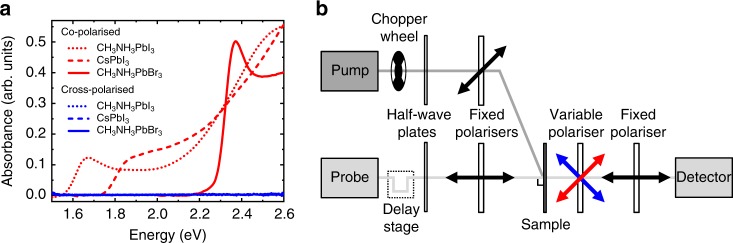
Sample properties and experimental setup. **a** Steady state linear absorption spectra of the investigated hybrid perovskite thin films. In co-polarized measurements (red), polarisers placed before and after the sample are aligned parallel. In cross-polarized measurements (blue), polarisers placed before and after the sample are aligned perpendicular. No intrinsic dichroism is observed. **b** Sketch of the experimental setup used for polarization selective transient absorption spectroscopy. The linear polarization of the pump pulse is set at an angle of 45° with respect to the linear polarization of the probe pulses before the sample. A second set of polarizers after the sample detects the linear polarization component of the probe pulses either parallel or perpendicular to the pump pulse polarization direction

Thin film lead halide perovskite samples were prepared using standard procedures^26^. CH_3_NH_3_PbI_3_ films were deposited on fused silica in nitrogen atmosphere by spin-coating from a 3:1 molar solution of CH_3_NH_3_I and either PbCl_2_ or Pb(CH_3_COO)_2_. CH_3_NH_3_PbI_3_ bulk films are continuous (thickness of approximately 300 nm) with a polycrystalline domain size of a few µm for the chloride precursor and a few 100 nm for the acetate precursor (Supplementary Figure 1). CsPbI_3_ nanocrystals were synthesized according to the procedure of Protesescu et al.^27^ with an average size of 14 nm and a cubic shape (Supplementary Figure 1), and thin films were prepared by spin-coating a 10 mg ml^−1^ nanocrystal solution onto fused silica at 2000 rpm. CH_3_NH_3_PbBr_3_ films were deposited on fused silica in nitrogen atmosphere by spin-coating from a 3:1 molar solution of CH_3_NH_3_Br and Pb(CH_3_COO)_2._ The optical properties of the thin films are consistent with previous literature reports, and we confirmed that the samples show no intrinsic optical anisotropy (Fig. 1a) using steady state linear absorption measurements. We estimate the optical band gap to be at 1.6 eV (CH_3_NH_3_PbI_3_), 1.8 eV (CsPbI_3_), and 2.3 eV (CH_3_NH_3_PbBr_3_). CdSe/CdS nanocrystals were prepared using the method of Bae et al.^28^ with diameter of 12 nm^29^ and optical band gap of 1.8 eV.

Samples were excited with linearly polarized pump pulses (pulse length less than 50 fs, approximately 25 meV FWHM) at energies between 10 and 200 meV above the optical bandgap and probed after a variable time delay with linearly polarized probe pulses with broad spectral range (1.55–2.55 eV). We selectively recorded the polarization component of the probe pulses aligned parallel or perpendicular to the linear polarization of the pump pulses directly after the sample. This arrangement avoids artifacts from polarization-dependent reflection or excitation intensity changes (Fig. 1b). Probe transmission spectra through the sample with (*T*_on_) and without (*T*_off_) the pump pulse are used to calculate the relative change in transmission following photoexcitation $\frac{{\Delta T}}{T} = \frac{{T_{{\mathrm{on}}} - T_{{\mathrm{off}}}}}{{T_{{\mathrm{off}}}}}$.

**Fig. 2:**
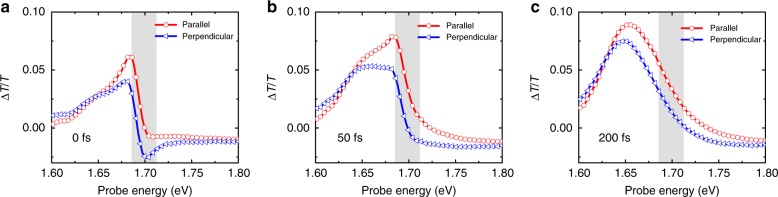
Ultrafast polarization selective transient absorption. **a**–**c** Spectrally resolved sub-picosecond transient absorption response of CH_3_NH_3_PbI_3_ hybrid perovskite thin films at the indicated time delays. The sample was excited with laser pulses of energy 1.7 eV (FWHM approximately 25 meV, shaded area), pulse length of approximately 50 fs, and excitation fluence approximately 20 µJ cm^−2^. The change in the transmission of the probe was measured at the indicated time delays with the linear polarization of the probe pulse aligned either parallel (red) or perpendicular (blue) to the linear polarization of the pump pulse. We observe a stronger increase in transmission (photoinduced bleach signal) when the pump and probe pulse polarizations are aligned parallel. The differences observed in the transient spectral dynamics indicate that the carrier populations probed in parallel and perpendicular configurations undergo different relaxation

Control samples of CdSe/CdS nanocrystals excited at 1.91 eV and probed between 1.78 and 1.91 eV show no detectable anisotropy in $\Delta T/T$ within our time-resolution, as expected (Supplementary Figure 2). Figure 2 presents the spectrally resolved values for $\Delta T/T$ for CH_3_NH_3_PbI_3_ films excited at 1.7 eV.

Immediately after photoexcitation (*t* = 0 fs, Fig. 2a) we observe a strong positive signal close to the excitation energy in both polarization configurations, representing a bleach of the ground-state transitions by a population of excited carriers. The weak tail of this bleach extending towards the band edge at 1.64 eV indicates that a small fraction of carriers has started to thermalize, while a large fraction of carriers remains non-thermal, i.e., these have not yet scattered with other carriers and phonons. The signal in the parallel configuration is stronger than in the perpendicular configuration, indicating that the parallel probe interacts with either a larger carrier population, or the excited carrier population exhibits a different TDM for different linear polarization directions. We take this as a first indication that the symmetry in the optical properties is broken by the linear polarization of the pump pulse. At later time delays (*t* = 50 fs, Fig. 2b), the signal in the perpendicular configuration is strongly broadened towards the band edge, which we take as a sign that these carriers have thermalized further and started to cool. Unexpectedly, the signal in parallel configuration remains rather localized at energies close to the excitation energy (gray area). This suggests that the carrier population probed in parallel configuration has relaxed less. The differences in signal intensity remain, and neither distributions have yet fully reached a thermalized distribution. By 200 fs (Fig. 2c), both parallel and perpendicular spectra evolve to spectral shapes well-described by thermalized Fermi-Dirac distributions and are now dominated by a strong positive signal close to the band edge, which has previously been assigned to phase-space filling by photoexcited carriers^5,30^. We note that the signal probed in perpendicular configuration maintains a different spectral shape and exhibits lower signal intensity by approximately 30%. These persistent differences in signal intensity and shape suggest that the initial anisotropy is preserved through thermalization and cooling processes, such as carrier–carrier and carrier-lattice scattering.

**Fig. 3:**
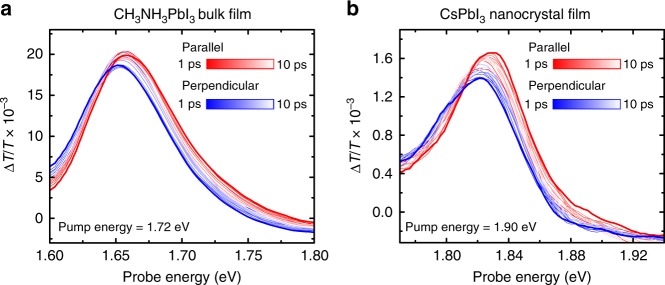
Spectrally resolved picosecond transient absorption spectra. **a** CH_3_NH_3_PbI_3_ hybrid perovskite bulk film (excitation approximately 1.72 eV, 15 µJ cm^−2^) and **b** CsPbI_3_ nanocrystal film (excitation approximately 1.90 eV, 4 µJ cm^−2^). The samples were excited with laser pulses of FWHM approximately 25 meV and pulse length of approximately 50 fs. The change in transmission of the probe was measured at the indicated time delays with the linear polarization of the probe pulse aligned parallel (red) or perpendicular (blue) to the linear polarization of the pump pulse. The initially polarization-dependent spectra converge within the first 10 ps after photoexcitation

We now discuss the anisotropy decay dynamics in the LP-TA of CH_3_NH_3_PbI_3_ over picosecond timescales in Fig. 3a. Two differences are apparent: The overall bleach intensity is higher for the parallel configuration, and the spectral shapes are different. Specifically, the bleach signal in the parallel configuration is higher on the high-energy side of the spectrum, while the opposite behavior is found on the low-energy side. The differences in spectral shape and intensity reduce over time, both converging to an intermediate spectrum with equal intensities by approximately 10 ps. We extract LP-TA kinetics at the center of the photoinduced bleach signal (1.64 eV in the CH_3_NH_3_PbI_3_ bulk film) to study the impact on population decay on the observed anisotropy. A linear superposition of parallel and perpendicular kinetics, equivalent to a magic angle pump-probe measurement, estimates the total population kinetics (Supplementary Figure 3). This value remains stable to longer than 10 ps, as expected at the excitation densities used, which indicates that negligible recombination occurs on the timescale over which the anisotropy is lost.

We observe similar anisotropy dynamics in CH_3_NH_3_PbI_3_ bulk films formed from both acetate and chloride precursors, and in CsPbI_3_ nanocrystal films (Fig. 3b, Supplementary Figure 3 and 4). At the excitation densities used, we only form one excitation on average per nanocrystal, which excludes interactions between multiple excited carriers as the origin of the anisotropy decay. Similar anisotropy effects are also observed for CH_3_NH_3_PbBr_3_ bulk films^31^ and CsPbBr_3_ bulk films (Supplementary Figures 5–7). We conclude that the polarization memory has a fundamental origin beyond the macroscopic crystal structure and is not determined by diffusion between crystallites^32^.

### Polarization anisotropy maps and dynamics

Polarization anisotropy maps give insight into spectral dynamics of the anisotropy. In accordance with previous literature, we use a standard definition of polarization anisotropy^33^ *A* as a function of time and probe energy from the difference between LP-TA signals obtained in parallel $\left( {\frac{{\Delta T}}{T}} \right)_\parallel$ or perpendicular $\left( {\frac{{\Delta T}}{T}} \right)_ \bot$ configurations:

1 $$A = \frac{{\left( {\frac{{\Delta T}}{T}} \right)_\parallel \,-\, \left( {\frac{{\Delta T}}{T}} \right)_ \bot }}{{\left( {\frac{{\Delta T}}{T}} \right)_\parallel \,+\, 2 \cdot \left( {\frac{{\Delta T}}{T}} \right)_ \bot }}$$

**Fig. 4:**
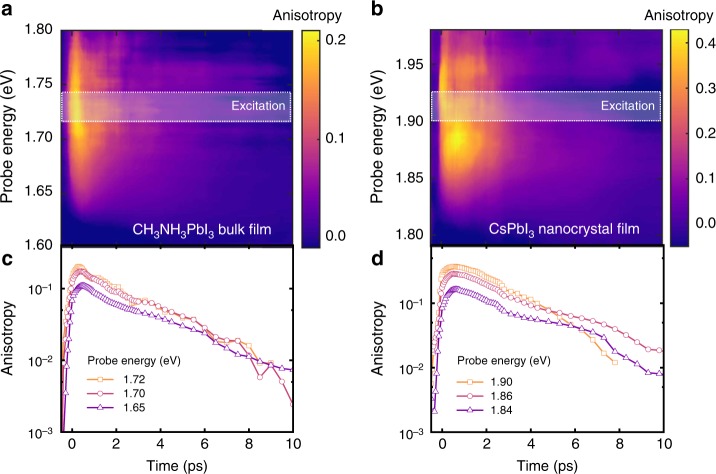
Spectrally and temporally resolved polarization anisotropy maps. **a** CH_3_NH_3_PbI_3_ hybrid perovskite bulk film (excitation at approximately 1.72 eV, 2 µJ cm^−2^, 50 fs) and **b** CsPbI_3_ nanocrystal film (excitation at approximately 1.90 eV, 3 µJ cm^−2^, 50 fs). A constant photoinduced absorption background was offset before calculating the polarization anisotropy. During the first 10 ps after photoexcitation, the polarization anisotropy shifts to states closer to the band minima. Polarization anisotropy decay kinetics of **c** CH_3_NH_3_PbI_3_ hybrid perovskite bulk film and **d** CsPbI_3_ nanocrystal film. After initial relaxation, the anisotropy signal decays exponentially with a time constant of 2.85 ± 0.1 ps in the CH_3_NH_3_PbI_3_ bulk film and 2.45 ± 0.2 ps in the CsPbI_3_ nanocrystal film. No significant variation in this decay time is found for different probe energies

Immediately after photoexcitation, the anisotropy is largest near the pump energy in both CH_3_NH_3_PbI_3_ thin films (Fig. 4a) and CsPbI_3_ nanocrystal films (Fig. 4b). Within the first picosecond after excitation, the anisotropy maximum relaxes towards lower energies, also visible by an increase in the anisotropy rise times (up to 150 fs) towards the band edge (Supplementary Figure 8). The following decay of the anisotropy after thermalization can be quantified from the anisotropy kinetics at selected probe energies (Fig. 4c, d). The signal follows a single-exponential decay with a lifetime of 2.85 ± 0.1 ps for the CH_3_NH_3_PbI_3_ bulk film and 2.45 ± 0.2 ps for the CsPbI_3_ nanocrystal film, respectively (Supplementary Figure 9). No significant variation in these decay times is found for other probe energies, which suggests that the loss of the photoexcited anisotropy occurs through a common process for all states. These timescales are much longer than typical phonon emission^34–36^ and carrier cooling times^5,30,37^, but are in agreement with reported timescales for dynamic fluctuations of the crystal lattice^8,38^. We take these observations as an indication that the origin for the persistent polarization anisotropy relates to the crystal conformation, rather than a particular carrier-phonon interaction. The similarity of the timescales, independent of crystallite size, suggests that the observed anisotropy is not lost through grain boundary scattering or diffusion between crystallites^32^, but through processes operating on considerably smaller length-scales.

**Fig. 5:**
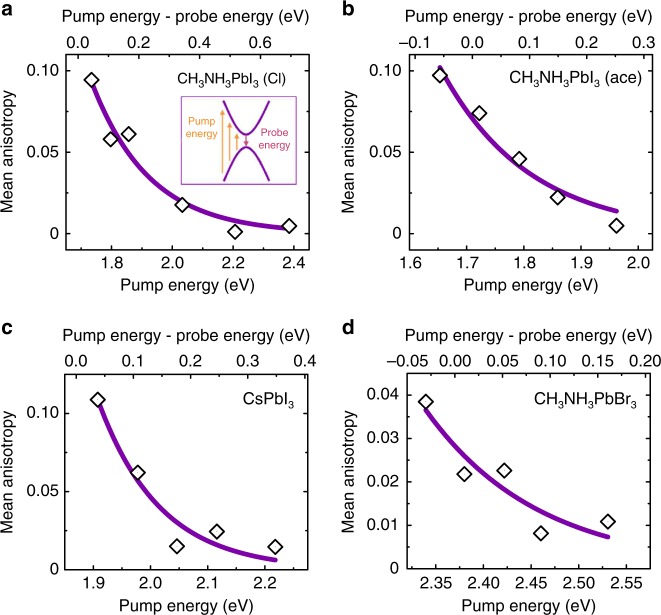
Change in polarization anisotropy with pump energy. **a** CH_3_NH_3_PbI_3_ bulk films prepared with chloride precursor, **b** CH_3_NH_3_PbI_3_ bulk films prepared with acetate precursor, **c** CsPbI_3_ nanocrystal films and **d** CH_3_NH_3_PbBr_3_ bulk films. The energy per pulse was approximately 2 µJ cm^−2^ (CH_3_NH_3_PbI_3_ Cl), 16 µJ cm^−2^ (CH_3_NH_3_PbI_3_ acetate), 40 µJ cm^−2^ (CsPbI_3_), and 1 µJ cm^−2^ (CH_3_NH_3_PbBr_3_). The mean anisotropy was defined as the temporal and spectral average of the polarization anisotropy over the first 10 ps (iodide samples) or first 1 ps (bromide sample), over a 40 meV probe window near the bleach maximum. The data is fitted with single exponentials, with decay constants of 190 meV (CH_3_NH_3_PbI_3_ Cl), 155 meV (CH_3_NH_3_PbI_3_ acetate), 110 meV (CsPbI_3_), and 120 meV (CH_3_NH_3_PbBr_3_)

Different pump energies create excited states at different positions in the band, which allows probing the dependence of the polarization anisotropy on the properties of the initially excited state (Fig. 5). The anisotropy is measured at a fixed energy near the peak of the photoinduced bleach (1.71 eV for CH_3_NH_3_PbI_3_ acetate films, 1.69 eV for CH_3_NH_3_PbI_3_ chloride films, 1.87 eV for CsPbI_3_ nanocrystal films, 2.37 eV for CH_3_NH_3_PbBr_3_ films) and averaged over the first 10 ps in the iodide samples and over the first 1 ps in the bromide sample. The pump energy was varied from 1.7 to 2.5 eV, while keeping the excitation density constant. We find that the anisotropy is largest for pump energies close to resonance with the probe energy, and it decreases as the difference between pump and probe energy increases. We fitted the pump energy dependence of the anisotropy with an exponential decay. The characteristic energies are approximately 150 meV (Fig. 5), which is over an order of magnitude higher than the dominant phonon modes of the material (approximately 50 cm^−1^ = 6 meV). In measurements with either increasing pump energies (Supplementary Figure 10) or increasing excitation density (Supplementary Figure 11), we observe a reduction in the polarization anisotropy peak value, but not its lifetime.

**Fig. 6:**
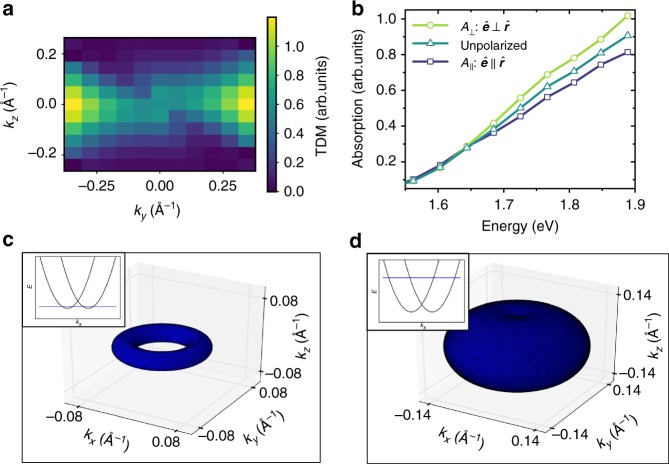
First-principles calculation of transition dipole moments (TDMs). **a** Plot of the TDM **k**-space asymmetry for energies close to the band minima. **b** Calculated absorption spectrum from first principles for electric field polarization direction $\hat e$ parallel and perpendicular to the Rashba direction of the crystal lattice $\hat r$, and for unpolarized light. Iso-energetic surfaces of photoexcited carrier distributions for photon energies **c** above and **d**  near the band edge. The insets show parabolic band disperisons and indictae the energies at which the iso-energetic **k**-space surfaces were calculated

To investigate the link between optically polarized excitation and probing, and the observed anisotropy, we turn to modeling and first principles calculations of the perovskite band structure (full details in Supplementary Methods 1 and 2). We consider a $\sqrt 2 \times \sqrt 2 \times 2$ unit cell of the tetragonal phase CH_3_NH_3_PbI_3_ structure. We use a structure with the MA molecules in a polar configuration which is known to be a local energy minimum for this unit cell^39^. This structure therefore serves as a local electronic structure model for CH_3_NH_3_PbI_3_. It has a polar axis in the *z*-direction, which breaks the local inversion symmetry. This causes spin-splitting of the conduction and valence bands, resulting in a Rashba-type band structure axially symmetric about the Rashba direction (here along the *z*-axis). Our calculations show that the breaking of the inversion symmetry has consequences not only for the band energies, but also for the transition dipole matrix elements. In Fig. 6a, b we plot the calculated TDM and ground state absorption close to the absorption edge. We find that the absorption is stronger when the light polarization axis is perpendicular to the Rashba direction, and weaker when it is parallel to the Rashba direction.

Given that no static polarization anisotropy is observable in our samples, we consider the local polarization of such nanoregions to be randomly oriented in space. We now discuss how excitation of such an isotropic distribution of polarized structures leads to overall polarization anisotropy. The theoretically calculated anisotropy in absorption is imprinted onto the carrier populations upon optical excitation. The total transient absorption signal for a sample containing many polarized nanoregions can be written in a condensed form as

2 $$\alpha = \mathop {\sum }\limits_{i \in {\mathrm{NR}}} {\mathrm{{\Pi}}}_i \times n_i,$$

where II_*i*_ is transition dipole matrix element squared in nanoregion *i*, and *n*_*i*_ is the density of available optical transitions in that nanoregion. Because each region has a different Rashba axis, II_*i*_ and *n*_*i*_ depend on nanoregion and cannot be treated as constant. The larger the TDMs along the light polarization axis, the more carriers will be excited by the pump, and the lower *n*_*i*_ will be. For the parallel and perpendicular configurations, the correlations between II_*i*_ and *n*_*i*_ will be different in magnitude. This leads to different values of *α* and generates the polarization anisotropy, as quantified below.

To understand how TDM anisotropy relates to polarization anisotropy, we first expand II and *n* as a series of spherical harmonics in Rashba direction and Brillouin zone **k**-vector. We then use these expansions to obtain expressions for transient absorption in parallel and perpendicular polarization configurations. This is done by integrating contributions to transient absorption and integrating over Brillouin zone **k**-vectors, and averaging over all possible Rashba directions, assumed isotropically distributed across nanoregions. Finally, we show that the polarization anisotropy can be written as

3 $$A = - \frac{3}{2}\frac{{C_{20}}}{{C_{00}}}\frac{{B_{20}}}{{B_{00}}}$$

where *C*_lm_ and *B*_lm_ are the coefficients of the expansions of II_*i*_ and *n*_*i*_ in spherical harmonics. We estimate values of *C*_lm_ from our DFT calculations by fitting ab-initio TDMs to a spherical harmonic expansion. The *B*_lm_ are likewise obtained from DFT calculations because the carrier densities induced by the pump are determined by the TDMs. We obtain a value of *A* = 0.09 for the polarization anisotropy of CH_3_NH_3_PbI_3_, for pump and probe energies of 1.7 eV. Comparing this theoretical estimate to the magnitudes of the maximum measured anisotropy immediately after excitation (Fig. 4) indicates that this mechanism is largely responsible for the observed polarization anisotropy. As this theoretical estimate is based on a single structure at a local energy minimum, it is likely that it will be increased if other non-equilibrium structures with higher polarity are considered as well. As the structure fluctuates on picosecond time scales, the Rashba axis changes stochastically in each nanoregion. We assume that this stochastic evolution approximates a random walk, with nanoregions losing memory of their initial Rashba directions after a characteristic time *τ*. Over this time scale, the values of II_*i*_ become randomized and uncorrelated with *n*_*i*_. The transient absorption signals for both parallel and perpendicular configurations would then regress towards the uncorrelated value $\left\langle \alpha \right\rangle = \left\langle {\mathrm{{\Pi}}} \right\rangle \times \left\langle {{n}} \right\rangle$ in the long-time limit, leading to a loss of polarization anisotropy, as in seen in Fig. 4.

Our theoretical model above assumes an isotropic distribution of carrier momenta shortly after photoexcitation—i.e., that thermalization and cooling processes randomize carrier momentum on approximately 200 fs time scales^40^. This is likely to hold for excitations with more than approximately 100 meV excess energy above the band edge, for which the excitation isosurface is approximately spherical (Fig. 6c, isosurface plot, high energy). However, excitations close to the band edge access a restricted momentum space, since the presence of polar distortions leads to toroidal isosurfaces (Fig. 6d, isosurface plot, low energy). To demonstrate how the changes in the momentum space distribution with energy affect transient absorption, we write the transient absorption signal for a single nanoregion _*i*_ as an integral over momentum space:

4 $$\alpha _i = {\int}_S {\mathrm d} ^3k\,{\mathrm{{\Pi}}}_i\left( {{ \overrightarrow {\mathbf k}}} \right)n_i(\overrightarrow {\mathbf k })$$

Here, the momentum resolved TDMs II*i*$(\overrightarrow {\mathbf k} )$ and the energy isosurface $S$ at the probe energy depend only on the probe energy and the Rashba direction. On the other hand, the momentum resolved carrier densities *n*_*i*_$(\overrightarrow {\mathbf k} )$ depend on the pump energy and the Rashba direction. As the pump energy is increased with respect to the band gap, the carrier density isosurface at excitation becomes progressively more spherical, and hence less correlated with the Rashba direction. This leads to decreasing correlation between *α*_*i*_ and Rashba direction with increasing separation between pump and probe energies. The dependence of polarization anisotropy on pump energy (Fig. 5) is consistent with this interpretation. However, we cannot rule out an additional influence from more energetic carriers losing excess energy to the lattice as they relax to the band edges, which could result in more rapid structural deformations of the lead-halide lattice and a partial randomization of the initial Rashba directions. Similarly, we cannot rule out a contribution from short-range diffusion of carriers between very small nanoregions, which may occur on similar timescales to structural deformation. However, the observed timescales and dependencies on pump and probe energy are difficult to reconcile with this process alone.

To model the energy dependence of transient absorption spectra, we insert the first-principles absorption spectra (Fig. 6b) into the analytical model for TA developed by Price et al. (Supplementary Method 3).^5^ This uses the Kramers–Kronig relations to consider the changes in both absorption and reflection caused by the excited carrier population, including state-filling via the Burstein–Moss effect and taking in to account Coulombic enhancement (Elliot’s model of absorption) and band-gap renormalization. Good qualitative agreement with experiment can be achieved in this way (Supplementary Figure 12A), although there is a mismatch in the crossing point of parallel and perpendicular polarizations. This crossing cannot be replicated by differences in the homogeneous and inhomogeneous broadening, reduced effective mass, or asymmetry in the effective masses of electrons and holes between the two experiments. A more quantitative fit can however be achieved if the effective carrier temperatures can differ for different polarizations (Supplementary Figure 12B). This recreates the experimental data more effectively, and highlights that there is different spectral broadening in the parallel and perpendicular spectra. This leads to the intriguing possibility that the parallel and perpendicular carrier populations interact only weakly, and maintain different temperatures, or carrier–phonon interactions, for several picoseconds. The origin of such an effect is beyond the scope of this investigation, but may well be justified given that carrier–phonon scattering rates have been shown to be dependent on carrier density and that diffusion effects between nanoregions are expected to dominate only at time scales longer than the cooling times.^5^

## Discussion

We report an optically excited picosecond transient anisotropy in the electronic states of lead halide perovskites. The picosecond timescale is orders of magnitude slower than the optical orientation observed in crystalline inorganic semiconductors^18^. Our results show that the anisotropy does not arise from the organic parts of the metal-halide perovskites. In agreement with modeling and first principles calculations, we conclude that the observed effects arise from dynamic structural anisotropies in the material that are sufficient to perturb the local band structure and accessible electronic states. This suggests that there is strong coupling between local anharmonic lattice dynamics^9^ and the optical properties of the electronic states in these materials, in contrast to classical crystalline semiconductors in which delocalized electronic states are rather insensitive to local lattice dynamics. Previous experiments and calculations suggest that dynamic deformations of small regions of the lead iodide lattice spontaneously lead to localized polarized distortions on picosecond timescales, with characteristic size of less than 10 nm^9,41^. Our work supports this interpretation, and we conclude that these distortions lead to carrier populations that interact only weakly over the timescales of lattice reorganization. We therefore consider each lead halide perovskite sample and crystallite to consist of multiple nanoregions, each with optical and electronic properties that dynamically fluctuate on picosecond timescales^8,15,42^ to create a dynamic landscape of electronic states. We suggest that these electronically distinct regions represent the fundamental units of perovskite electronic structure, rather than crystal domains or individual nanocrystals. Stabilizing the perovskite structure is therefore likely to increase the polarization anisotropy magnitude and lifetime. A possible mechanism is the application of suitable external strain onto the crystal lattice, for example through piezoelectric force. The soft nature of both hybrid and purely inorganic perovskites gives rise to behavior not observed in either classic organic or inorganic semiconductors, which has far-reaching implications for the understanding and application of this important class of materials.

## Methods

### Steady state linear absorption

Steady state linear absorption measurements were performed using a custom-modified Zeiss Axio A1 optical microscope equipped with a digital CCD camera (IDS UI-3580LE). Each sample was illuminated in transmission from a halogen lamp (Zeiss HAL100). A linear polarizer (Zeiss A1 polarizer D 427706) in the illumination beam path defined the orientation of the incident polarization. The incident light was focused through a condenser onto the sample (Zeiss 424225-9001). The transmitted light was coupled into a ×05, NA 0.13 objective (Zeiss EC Epiplan-Neofluar 1156-514). The reflected signal from the sample was filtered by a broadband wire grid linear polarizer (Thorlabs WP25M-UB). The polarizer was mounted on a motorized rotation stage on the optical path and rotated between the orientation parallel to the incident beam polarizer, and perpendicular to it. The central area of Ø300 μm reflected from the illuminated area was coupled into a 600 μm core optical fiber (Avantes FC-UV600) mounted in confocal configuration and measured in a spectrometer (Avantes Avaspec HS2048). The sample was manually rotated by 90° to check for evidence of static Kerr rotation.

### Linear polarization sensitive transient absorption

The output of a Ti:Sapphire amplifier system (Spectra-Physics Solstice) operating at 1 kHz and generating 90-fs pulses was split into the pump and probe beam paths. The visible broadband probe beams were generated in home-built noncollinear optical parametric amplifiers. The visible narrowband (25 meV full-width at half-maximum) pump beam was provided by a TOPAS optical parametric amplifier (Light Conversion). The transmitted pulses were collected with an InGaAs dual-line array detector (Hamamatsu G11608-512) driven and read out by a custom-built board from Stresing Entwicklungsbüro. The linear polarization of the pump beam was set at 45° with respect to the probe beam before the sample. During the measurement none of the polarizers before the sample were varied. Polarization dependent measurements were carried out according to Tan et al.^33^ by rotating a linear polarizer located in the probe beam immediately after the sample. This polarizer was set to be either parallel or perpendicular to the pump polarization. A final linear polarizer fixed at 45° to the variable polarizer was placed directly in front of the detector, in order to remove the effect of any detector polarization dependence. The chirp in all measurements were corrected by reference measurements of the coherent artifact on glass substrates. The chirp was corrected identically in the parallel and perpendicular measurements.

### Data availability

All relevant data is available on the Cambridge Repository at the following link: 10.17863/CAM.26301.

## Electronic supplementary material

Supplementary Information
