# Supplementary material for: Long-lived polarization memory in the electronic states of lead-halide perovskites from local structural dynamics
Source: Nat Commun. 2018 Aug 30;9:3531. doi: 10.1038/s41467-018-06009-3 (PMC6117347; doi:10.1038/s41467-018-06009-3)
Supplement: Supplementary file 1 — Supplementary Information [file 41467_2018_6009_MOESM1_ESM.pdf]

Supporting Material: Long-lived polarization memory in the electronic states of lead-halide perovskites from local structural dynamics

Jasmine P. H. Rivett[1], Liang Z. Tan[2], Michael B. Price[1], Sean Bourelle[1], Nathaniel J. L. K. Davis[3], James Xiao[1], Yatao Zou[5], Rox Middleton[4], Baoquan Sun[5], Andrew M. Rappe[2], Dan Credgington[1,*], Felix Deschler[1,*]

[1]Cavendish Laboratory, University of Cambridge, JJ Thomson Avenue, CB3 0HE Cambridge, UK

[2]Department of Chemistry, University of Pennsylvania, Philadelphia, Pennsylvania 19104, United States

[3]School of Chemical and Physical Sciences, Victoria University of Wellington, Wellington 6140, New Zealand

[4]Department of Chemistry, University of Cambridge, Lensfield Road, CB2 1EW Cambridge, United Kingdom

[5]Institute of Functional Nano & Soft Materials (FUNSOM), Soochow University, 199 Ren'ai Road, Suzhou 215123, People's Republic of China

Supplementary Figures

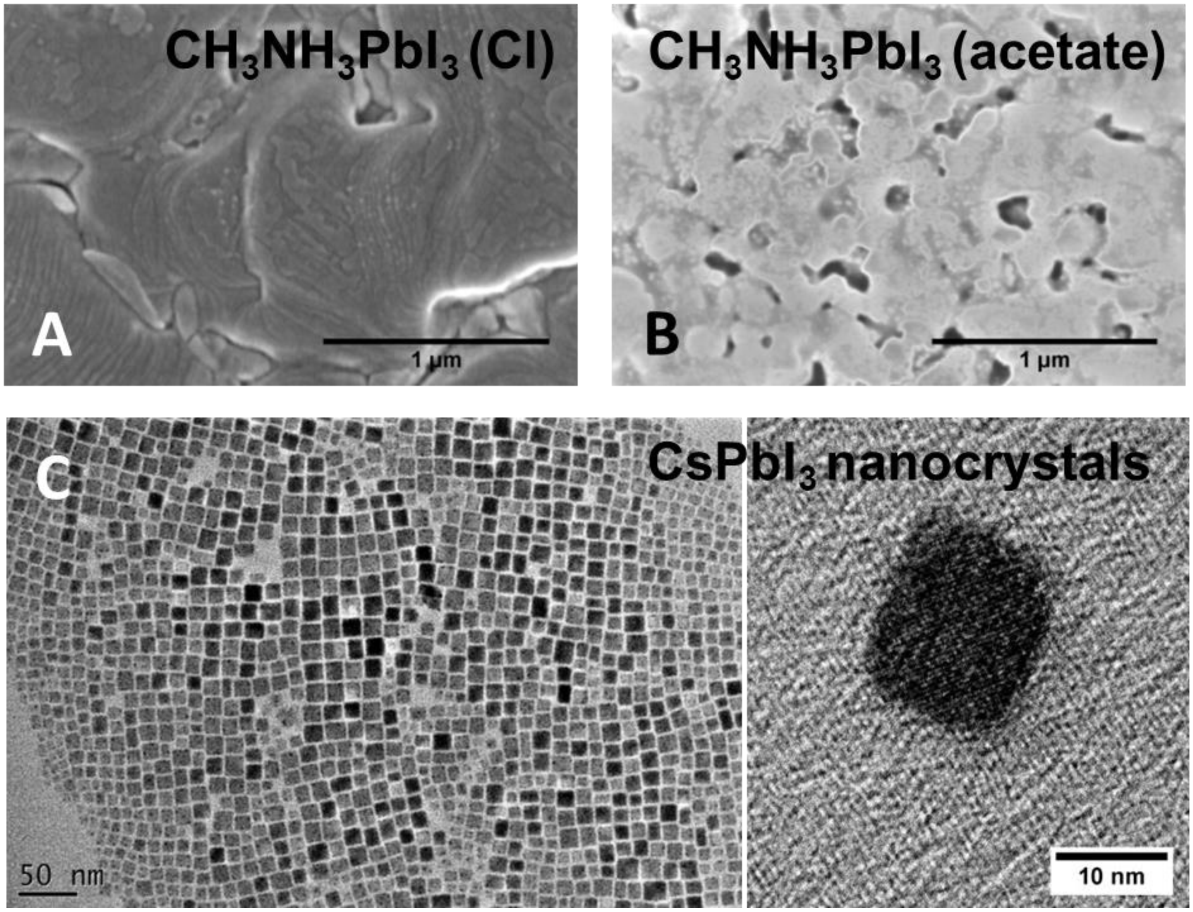

Supplementary Figure 1: Scanning electron microscope images of (A) $CH_3NH_3PbI_3$ bulk films prepared with chloride precursor, (B) $CH_3NH_3PbI_3$ bulk films prepared with acetate precursor. The films have a polycrystalline domain size of a few micrometers for the chloride precursor and several 100 nm for the acetate precursor. Transmission electron microscope images of (C) $CsPbI_3$ nanocrystals with an average size of approximately 14 nm and cubic shape.

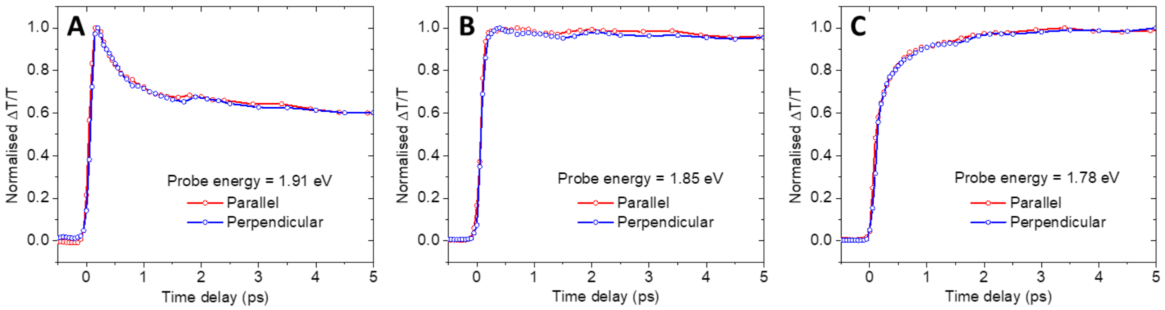

Supplementary Figure 2: Ultrafast polarization selective transient absorption kinetics of CdSe/CdS nanocrystals thin film (A-C) with ground state bleach peak at 1.85eV. Temporally resolved transient absorption response of CdSe/CdS nanocrystals thin film at the indicated probe energies. The sample was excited with laser pulses of energy 1.91 eV (FWHM approximately 25 meV), pulse length of approximately 100 fs, and excitation fluence approximately 10 μJ cm^{-2}. The change in the transmission of the probe was measured at the indicated probe energies with the linear polarization of the probe pulse aligned either parallel (red) or perpendicular (blue) to the linear polarization of the pump pulse. We observe that the data obtained with the pump and probe polarizations aligned parallel or perpendicular follow the same kinetics, confirming the working mechanism of our experimental setup.

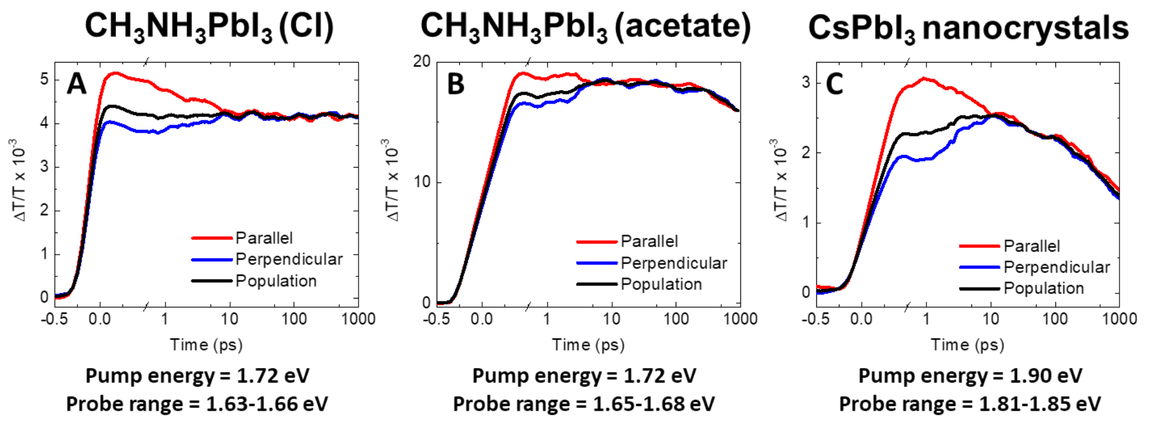

Supplementary Figure 3: Kinetics extracted from polarization selective transient absorption of (A) $CH_3NH_3PbI_3$ bulk films prepared using chloride precursor, (B) $CH_3NH_3PbI_3$ bulk films prepared using acetate precursor, (C) $CsPbI_3$ nanocrystal films. Kinetics were measured with the linear polarization of the probe pulse aligned either parallel (red) or perpendicular (blue) to the linear polarization of the pump pulse. The population kinetics (black) were calculated from the parallel $\left(\frac{\Delta T}{T}\right)_{\parallel}$ and perpendicular $\left(\frac{\Delta T}{T}\right)_{\perp}$ data using the formula $\frac{\left(\frac{\Delta T}{T}\right)_{\parallel} + 2\left(\frac{\Delta T}{T}\right)_{\perp}}{3}$. The initially polarization-dependent kinetics converge within the first 10 ps after photoexcitation. The energy per pulse was approximately 2 μJ cm^{-2} ($CH_3NH_3PbI_3$ Cl), 16 μJ cm^{-2} ($CH_3NH_3PbI_3$ acetate) and 3 μJ cm^{-2} ($CsPbI_3$).

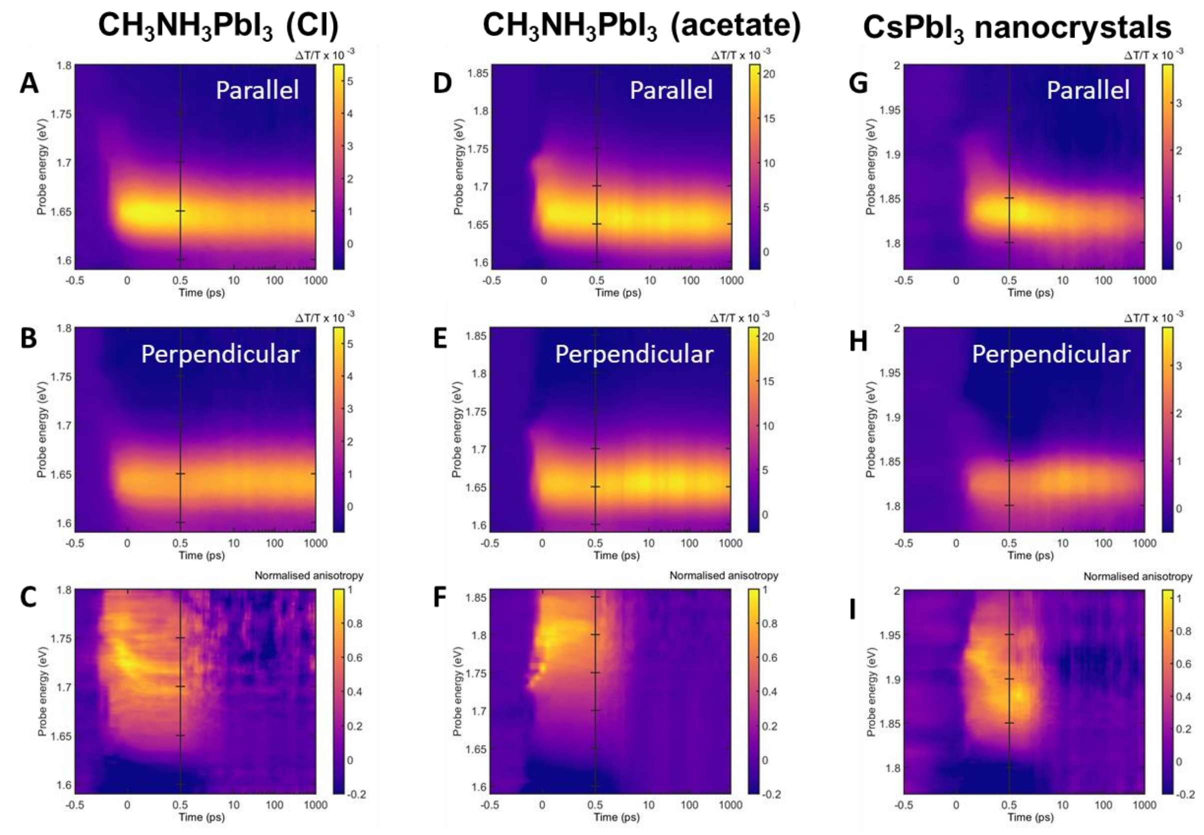

Supplementary Figure 4: Maps of the polarization selective transient absorption on (A-B) $CH_3NH_3PbI_3$ bulk films prepared using chloride precursor, (D-E) $CH_3NH_3PbI_3$ bulk films prepared using acetate precursor, (G-H) $CsPbI_3$ nanocrystal films. (C, F, I) Maps of the corresponding polarization anisotropy. The energy per pulse was approximately 2 μJ cm^{-2} ($CH_3NH_3PbI_3$ Cl), 16 μJ cm^{-2} ($CH_3NH_3PbI_3$ acetate) and 3 μJ cm^{-2} ($CsPbI_3$).

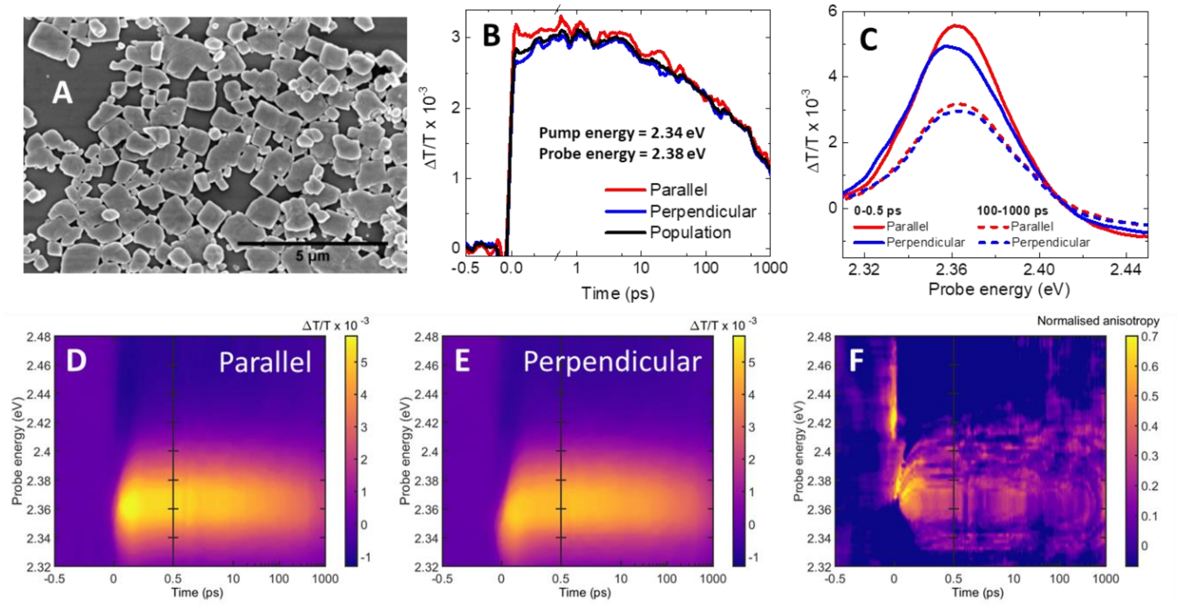

Supplementary Figure 5: (A) Scanning electron microscope image of a $CH_3NH_3PbBr_3$ bulk film prepared with acetate precursor. The films have a polycrystalline domain size of several 100 nm. (B-C) Kinetics and spectra extracted from polarization selective transient absorption of $CH_3NH_3PbBr_3$ bulk films prepared with acetate precursor. Kinetics and spectra were measured with the linear polarization of the probe pulse aligned either parallel (red) or perpendicular (blue) to the linear polarization of the pump pulse. The population kinetics and spectra (black) were calculated from the parallel $\left(\frac{\Delta T}{T}\right)_{\parallel}$ and perpendicular $\left(\frac{\Delta T}{T}\right)_{\perp}$ data using the formula $\frac{\left(\frac{\Delta T}{T}\right)_{\parallel} + 2\left(\frac{\Delta T}{T}\right)_{\perp}}{3}$. (D-F) Corresponding maps. The excitation energy per pulse was approximately 1 μJ cm^{-2}.

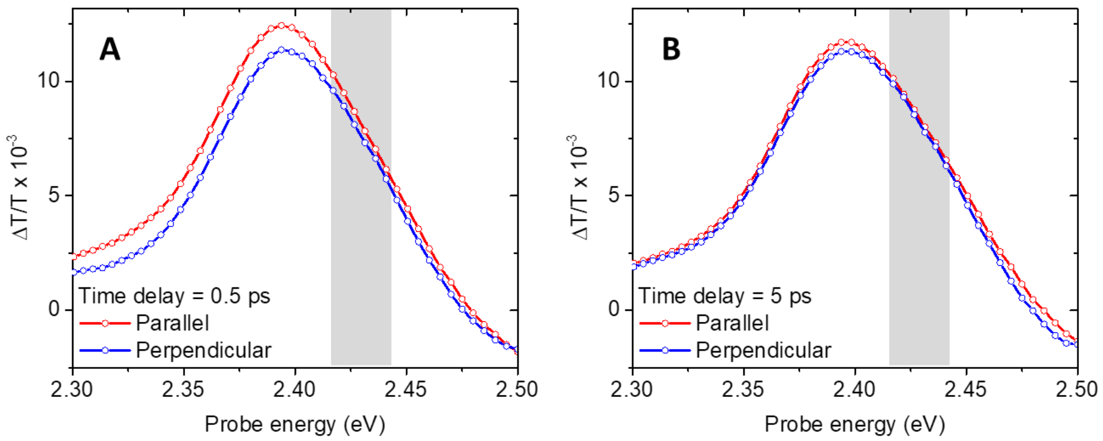

Supplementary Figure 6 Ultrafast polarization selective transient absorption spectra of bulk $CsPbBr_3$ thin film (A, B). Spectrally resolved transient absorption response of bulk $CsPbBr_3$ perovskite thin film at the indicated time delays. The sample was excited with laser pulses of energy 2.43 eV (FWHM approximately 25 meV, shaded area), pulse length of approximately 100 fs, and excitation fluence approximately 10 μJ cm^{-2}. The change in the transmission of the probe was measured at the indicated time delays with the linear polarization of the probe pulse aligned either parallel (red) or perpendicular (blue) to the linear polarization of the pump pulse. We observe a stronger increase in transmission (photoinduced bleach signal) when the pump and probe pulse polarizations are aligned parallel.

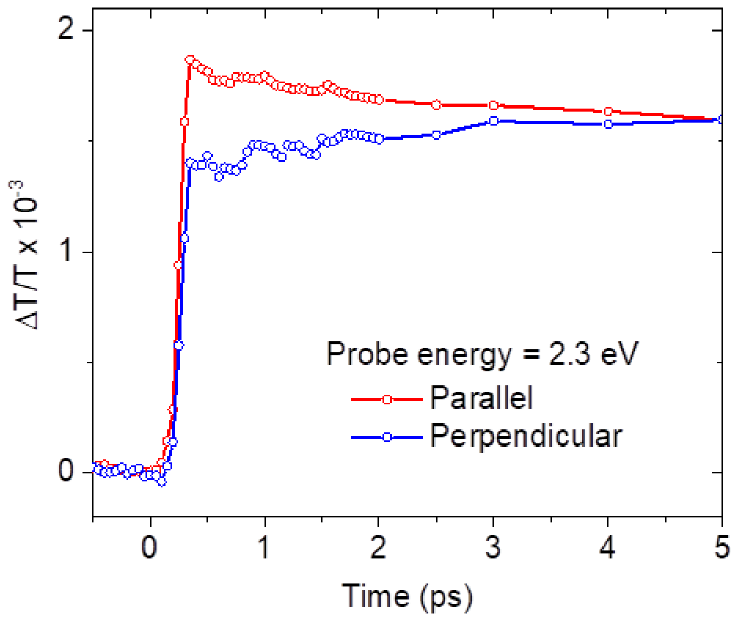

Supplementary Figure 7 Ultrafast polarization selective transient absorption kinetics of bulk $CsPbBr_3$ thin film. Temporally resolved transient absorption response of bulk $CsPbBr_3$ perovskite thin film at the indicated probe energy. The sample was excited with laser pulses of energy 2.43 eV (FWHM approximately 25 meV), pulse length of approximately 100 fs, and excitation fluence approximately 10 µJ cm^{-2}. The change in the transmission of the probe was measured at the indicated probe energy with the linear polarization of the probe pulse aligned either parallel (red) or perpendicular (blue) to the linear polarization of the pump pulse. We observe a stronger increase in transmission (photoinduced bleach signal) when the pump and probe pulse polarizations are aligned parallel.

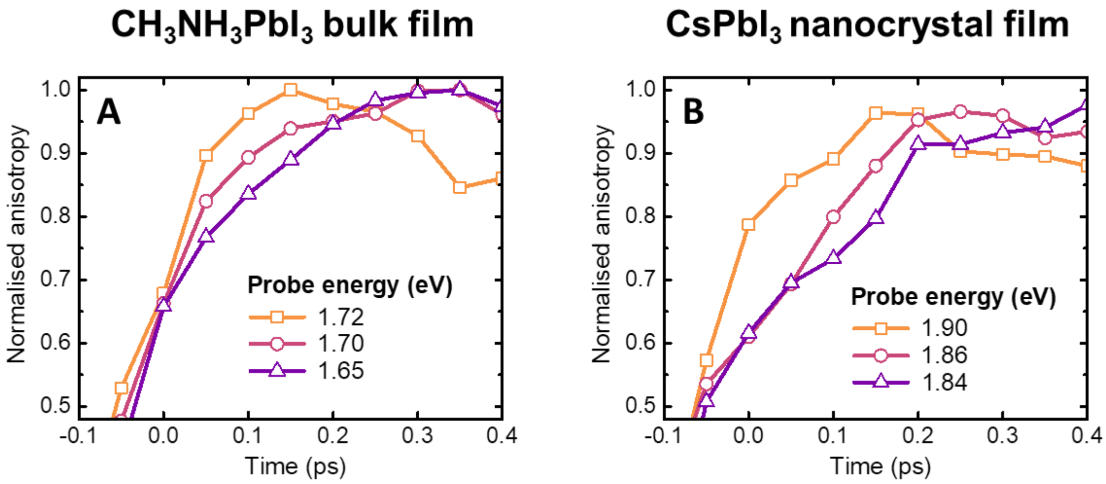

Supplementary Figure 8: Rise in anisotropy kinetics of (A) $CH_3NH_3PbI_3$ bulk perovskite and (B) $CsPbI_3$ nanocrystals at the specified probe energies. Excitation was performed at 1.72 eV (1 kHz, 100 fs, 2 μJ cm^{-2}) on $CH_3NH_3PbI_3$ bulk perovskite and 1.90 eV (1 kHz, 100 fs, 3 μJ cm^{-2}) on $CsPbI_3$ nanocrystals. The signal shows a slower rise for lower probe energies below the pump energy.

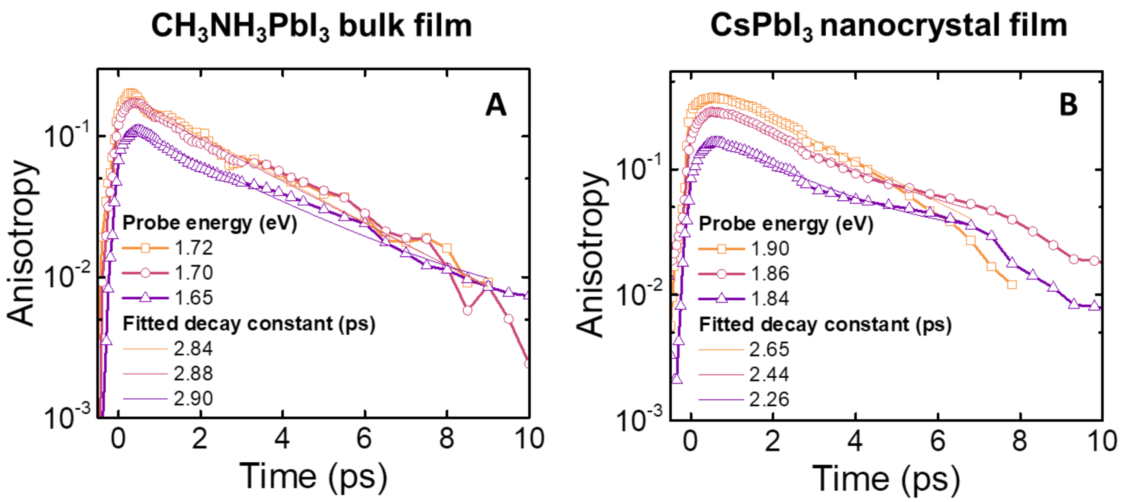

Supplementary Figure 9: Fits to the evolution of the transient absorption polarization anisotropy in the first picoseconds after excitation in (A) $CH_3NH_3PbI_3$ bulk thin films and (B) $CsPbI_3$nanocrystal films. The fits are single exponential decays, with lifetimes as indicated. No significant variation in the fitted decay time is found for different probe energies. The energy per pulse was approximately 2 μJ cm^{-2} (bulk film) and 3 μJ cm^{-2} (nanocrystal film).

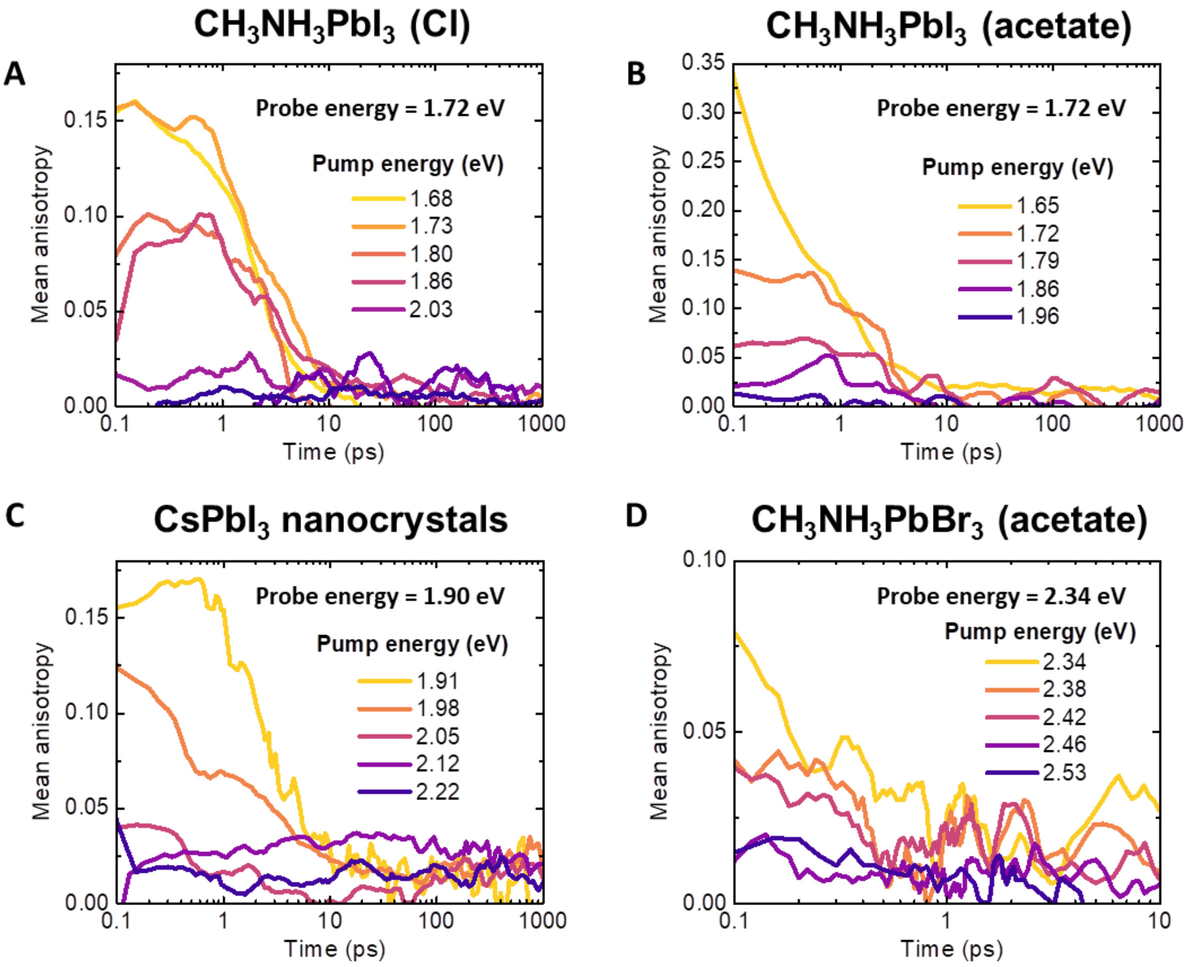

Supplementary Figure 10: Polarization anisotropy pump energy dependence. Kinetics of the mean anisotropy measured using polarization selective transient absorption. The anisotropy decays within the first 10 ps in all iodide samples and within 1 ps in the bromide sample. The energy per pulse was approximately 2 μJ cm^{-2} ($CH_3NH_3PbI_3$ Cl), 16 μJ cm^{-2} ($CH_3NH_3PbI_3$ acetate), 40 μJ cm^{-2} ($CsPbI_3$) and 1 μJ cm^{-2} ($CH_3NH_3PbBr_3$).

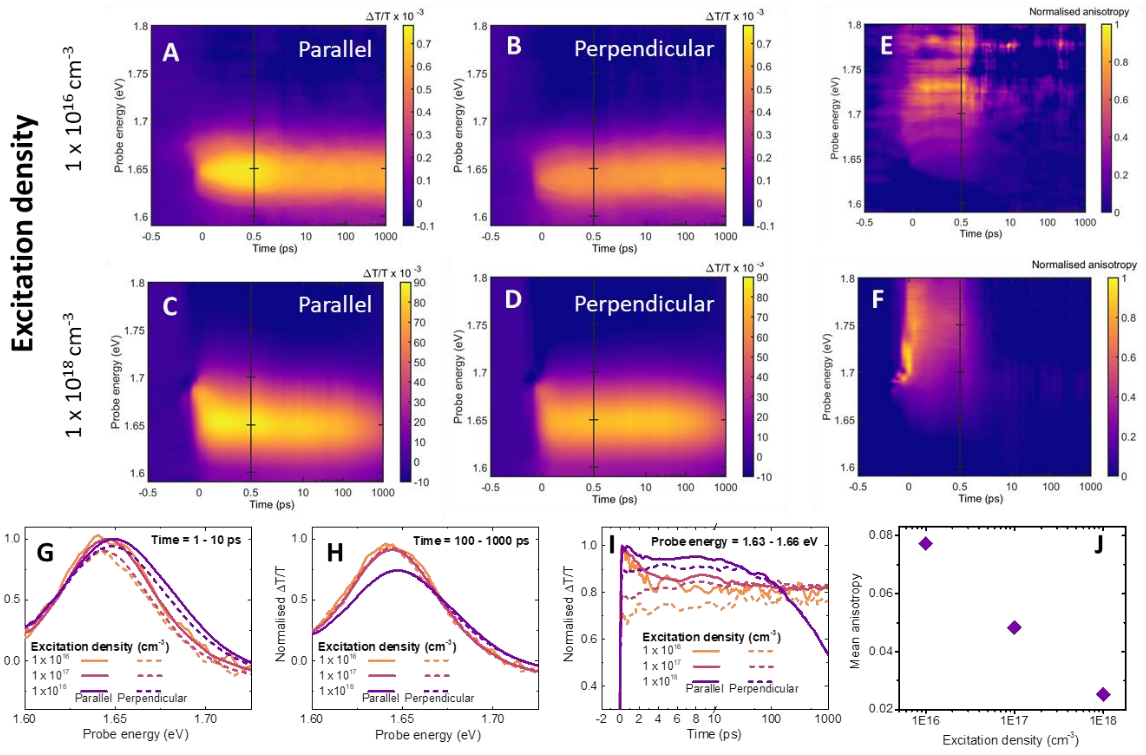

Supplementary Figure 11: Maps of the polarization selective transient absorption on $CH_3NH_3PbI_3$ bulk films $CH_3NH_3PbI_3$ bulk films (A-D) and maps of the corresponding polarization anisotropy (E,F). Samples were excited at approximately 1.72 eV, 1 kHz, 100 fs. (G,H) TA spectra near the band edge at different time delays and excitation densities. (I) TA kinetics near the absorption onset for different excitation energies. (J) Mean polarization anisotropy from 0 to 10 ps over the spectral range 1.64 to 1.68 eV for different excitation energies.

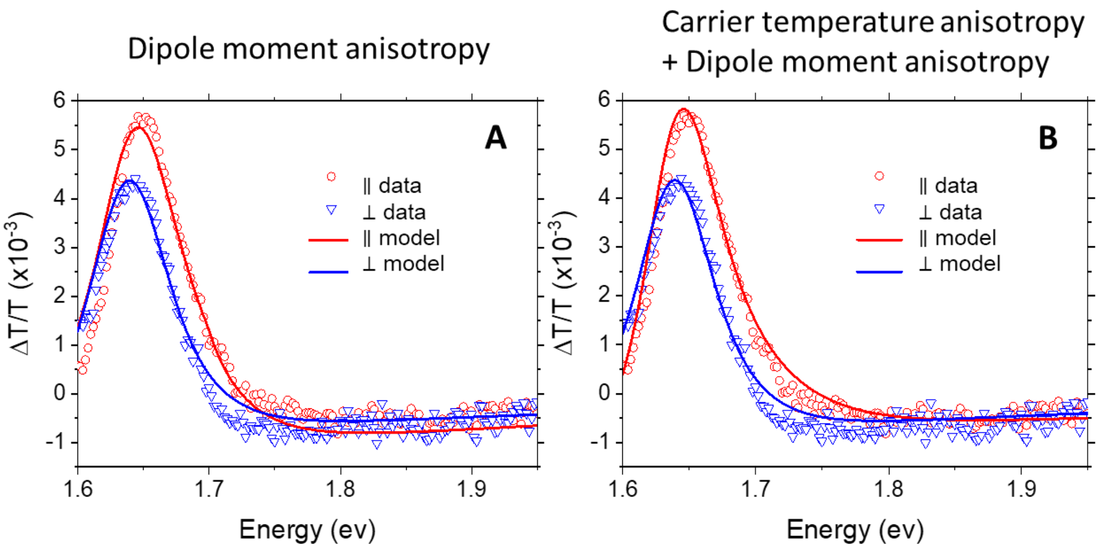

Supplementary Figure 12 Experimental TA spectra of $CH_3NH_3PbI_3$ bulk perovskite (pump approximately 1.72 eV, 1 kHz, 100 fs, 2.0 uJ cm^{-2} at t = 4 ps, hollow circles and triangles) plotted with (A) Modelled TA spectra (solid lines). Blue shows the spectra for pump perpendicular to probe, and red shows the parallel configuration. The spectra are modelled as per Price *et al.* [1], with parameters held constant except for the transition dipole moment which is given an anisotropy as in Figure 6. See methods section above for model parameters. (B) Modelled TA spectra including a change in effective carrier temperature in addition to the anisotropic transition dipole moment. The parallel spectrum has an effective carrier temperature of 410 K, compared to 310 K for the perpendicular spectrum.

Supplementary Methods

Supplementary Method 1, Anisotropy calculations using polar nanoregion model

The transient absorption at energy E and time t, with pump polarization direction $\hat{\boldsymbol{e}}_{pump}$ and probe direction $\hat{\boldsymbol{e}}_{probe}$, in a nanoregion with Rashba axis $\hat{\boldsymbol{r}}$, is

$$\alpha\left(E, \hat{\boldsymbol{r}}, \hat{\boldsymbol{e}}_{pump}, \hat{\boldsymbol{e}}_{probe}, t\right) = \sum_{\Omega_k} \Pi\left(\hat{\boldsymbol{r}}, \hat{\boldsymbol{e}}_{probe}, t, \vec{\boldsymbol{k}}\right) n\left(\hat{\boldsymbol{r}}, \hat{\boldsymbol{e}}_{pump}, t, \vec{\boldsymbol{k}}\right)$$

where the sum is over the solid angle of all **k**-points on the constant energy surface E. Here, $\Pi\left(\hat{\boldsymbol{r}}, \hat{\boldsymbol{e}}_{probe}, t, \vec{\boldsymbol{k}}\right) = \left|\langle c|\vec{\boldsymbol{p}} \cdot \vec{\boldsymbol{A}}_{probe}|v\rangle\right|^2$ is the transition dipole element squared at **k**-point $\vec{\boldsymbol{k}}$, and $n\left(\hat{\boldsymbol{r}}, \hat{\boldsymbol{e}}_{pump}, t, \vec{\boldsymbol{k}}\right) = \left(1 - f_c\left(\hat{\boldsymbol{r}}, \hat{\boldsymbol{e}}_{pump}, t, \vec{\boldsymbol{k}}\right)\right)\left(1 - f_v\left(\hat{\boldsymbol{r}}, \hat{\boldsymbol{e}}_{pump}, t, \vec{\boldsymbol{k}}\right)\right)$ is the density of available optical transitions at **k**-point $\vec{\boldsymbol{k}}$. Here, $\vec{\boldsymbol{A}}_{probe}$ is the probe vector potential with polarization along $\hat{\boldsymbol{e}}_{probe}$, and f_c and f_v are the fractional occupation of the conduction and valence bands with carriers at **k**-point $\vec{\boldsymbol{k}}$. The time dependence of Π and n arise from dynamic evolution of the structure away from the initial structure at the instant of pumping. The TDMs Π are dependent on the Rashba direction, as are the band structure and wavefunctions. The density of transitions n is Rashba direction dependent because the anisotropy of the initial carrier density is Rashba direction dependent. The total absorption of a macroscopic sample is an average over local nanoregions with varying Rashba directions

$$\langle\alpha\left(E, \hat{\boldsymbol{e}}_{pump}, \hat{\boldsymbol{e}}_{probe}, t\right)\rangle = \sum_{\Omega_k \Omega_r} \Pi\left(\hat{\boldsymbol{r}}, \hat{\boldsymbol{e}}_{probe}, t, \vec{\boldsymbol{k}}\right) n\left(\hat{\boldsymbol{r}}, \hat{\boldsymbol{e}}_{pump}, t, \vec{\boldsymbol{k}}\right)$$

The transition dipole elements for $\vec{\boldsymbol{k}}$ on the energy isosurface E may be expanded as series in spherical harmonics in both the Rashba direction and the **k**-vector direction

$$\Pi\left(\hat{\boldsymbol{r}}, \hat{\boldsymbol{e}}_{probe}, t, \vec{\boldsymbol{k}}\right) = \sum_{l_r m_r; l_k m_k} C_{l_r m_r; l_k m_k}\left(E, \hat{\boldsymbol{e}}_{probe}, t\right) Y_{l_r m_r}(\Omega_r) Y_{l_k m_k}(\Omega_k)$$

Here, the Y_{lm} are referenced to the lab-frame z-axis: Ω_r is the direction of the Rashba axis relative to the z-axis, and Ω_k is the direction of the **k**-vector relative to the z-axis. The angular momentum $l_r m_r$ and $l_k m_k$ correspond to the Rashba direction and **k**-vector, respectively. The

coefficient $C_{00;00}$ is independent of $\hat{\boldsymbol{e}}_{probe}$, as this term has zero angular momentum. In general, the C coefficients transform under rotation as

$$C_{l_r m_r;l_k m_k}(E, \hat{\boldsymbol{e}}_{probe}, t) = \sum_{m_r' m_k'} D^{l_r}_{m_r' m_r}(R)^* D^{l_k}_{m_k' m_k}(R)^* \, C_{l_r m_r';l_k m_k'}(E, R\hat{\boldsymbol{e}}_{probe}, t)$$

where R is any rotation operator in the full rotation group and the D's are Wigner D-matrices. The density of transitions for $\vec{\boldsymbol{k}}$ on the energy isosurface E can similarly be written in terms of spherical harmonics

$$n(\hat{\boldsymbol{r}}, \hat{\boldsymbol{e}}_{pump}, t, \vec{\boldsymbol{k}}) = \sum_{l_r m_r;l_k m_k} B_{l_r m_r;l_k m_k}(E, \hat{\boldsymbol{e}}_{pump}, t) Y_{l_r m_r}(\Omega_r) Y_{l_k m_k}(\Omega_k)$$

The components with odd values of l_r, l_k are expected to vanish because of time reversal symmetry. Since electron-phonon scattering rates in the halide perovskites occur faster than the 0.1 ps time scale, we assume that carrier distributions are isotropic in $\vec{\boldsymbol{k}}$ over the ps time scales relevant in the transient absorption measurement. Therefore, we consider only the $l_k = 0$ components. Under these conditions, and taking $\hat{\boldsymbol{e}}_{pump}$ along the lab-frame z axis, the lowest order expansion for the density of transitions is then

$$n(E, \hat{\boldsymbol{r}}, \hat{\boldsymbol{e}}_{pump} = \hat{\boldsymbol{z}}, t, \vec{\boldsymbol{k}}) = B_{00;00} Y_{00}(\Omega_r) Y_{00}(\Omega_k) + B_{20;00} Y_{20}(\Omega_r) Y_{00}(\Omega_k)$$

$\hat{\boldsymbol{e}}_{probe}$ is along the z axis in the parallel configuration, while it is along the x axis in the perpendicular direction. Integrating over Ω_r and Ω_k, the transient absorption in the parallel and perpendicular directions is found to be

$$\alpha_{\parallel} = C_{00;00}(E, \hat{\boldsymbol{z}}, t) B_{00;00}(E, \hat{\boldsymbol{z}}, t) + C_{20;00}(E, \hat{\boldsymbol{z}}, t) B_{20;00}(E, \hat{\boldsymbol{z}}, t)$$

$$\alpha_{\perp} = C_{00;00}(E, \hat{\boldsymbol{x}}, t) B_{00;00}(E, \hat{\boldsymbol{z}}, t) + C_{20;00}(E, \hat{\boldsymbol{x}}, t) B_{20;00}(E, \hat{\boldsymbol{z}}, t)$$

Using the definition of polarization anisotropy (Equation 1 of main text), and the transformation rule for C above (to write $C_{20;00}(E, \hat{\boldsymbol{x}}, t)$ in terms of $C_{20;00}(E, \hat{\boldsymbol{z}}, t)$), we obtain

$$A = -\frac{3}{2} \frac{C_{20;00}}{C_{00;00}} \frac{B_{20;00}}{B_{00;00}}$$

which is Equation 3 of the main text.

Supplementary Method 2, Computational parameters for DFT calculations

We performed density functional theory calculations of the absorption spectrum using the PBE density functional [2] and norm-conserving pseudopotentials [3], in a planewave basis set with kinetic energy cutoff of 60 Ry. Spin-orbit coupling was included at a full relativistic level. A Monkhorst-Pack [4] grid of 4x4x4 k-points was used for self-consistent evaluation of the charge density, while a k-point grid with an effective density of 40x40x40 was used for calculation of the absorption spectrum. These calculations were performed with the Quantum Espresso software package [5].

Supplementary Method 3, Analytical model for transient absorption spectra

The analytical model developed by Price *et al.* [1] reproduces experimental TA spectra by first calculating an expected change in absorption caused by the state filling of a Coulombically enhanced density of states assuming approximately parabolic bands (from Elliot's theory for semiconductor absorption). A small red shift is included, arising from band-gap renormalization, along with a free-carrier absorption term. The subsequent absorption is convolved with a Gaussian broadening parameter. Applying the Kramers-Kronig relations to this absorption, and taking account of the reflections and interference from the perovskite interfaces, gives a physically justified model of the transient transmission signal ($\Delta T/T$), which fits well to experiment. The parameters used to model the data in Figure S9A are: carrier density = 2.33×10^{23} m^{-3}, band gap = 1.6245 eV, reduced effective mass = 0.18 m_e, effective mass asymmetry = 3, Gaussian broadening parameter = 24 meV, band gap renormalisation red shift = 10 meV, exciton binding energy = 16 meV and carrier temperature = 310 K with the transition dipole matrix varying according to the data calculated from Fig 6B. For Supplementary Figure 9B, all parameters are the same as for Supplementary Figure 9A, with the exception of effective carrier temperature: 410 K for parallel, 310 K for perpendicular. The absorption constant is also adjusted slightly to compensate for the broader distribution: parallel = 7.42×10^{12} $cms^{-1/2}$, perpendicular = 7.40×10^{12} $cms^{-1/2}$.

Supplementary References

1. Price, M. B., Hot-carrier cooling and photoinduced refractive index changes in organic-inorganic lead halide perovskites, *Nat. Commun.* **6**, 8420 (2015).
2. Perdew, J. P., Burke, K. & Ernzerhof, M. Generalized Gradient Approximation Made Simple, *Phys. Rev. Lett.***77,** 3865–8 (1996).
3. Rappe, A. M., Rabe, K. M., Kaxiras, E. & Joannopoulos, J. D. Optimized Pseudopotentials, *Phys. Rev. B Rapid Comm.* **41,** 1227–30 (1990).
4. Monkhorst, H. J. & Pack, J. D. Special points for Brillouin-zone integrations, *Phys. Rev. B* **13,** 5188–5192 (1976).
5. Giannozzi, P. *et al.*, QUANTUM ESPRESSO: a modular and open-source software project for quantum simulations of materials, *J. Phys.: Condens. Matter* **21,** 395502 (2009).
